# The health and cost burden of antibiotic resistant and susceptible *Escherichia coli* bacteraemia in the English hospital setting: A national retrospective cohort study

**DOI:** 10.1371/journal.pone.0221944

**Published:** 2019-09-10

**Authors:** Nichola R. Naylor, Koen B. Pouwels, Russell Hope, Nathan Green, Katherine L. Henderson, Gwenan M. Knight, Rifat Atun, Julie V. Robotham, Sarah R. Deeny

**Affiliations:** 1 National Institute for Health Research Health Protection Research Unit in Healthcare Associated Infection and Antimicrobial Resistance at Imperial College London, London, England; 2 Modelling and Economics Unit, National Infection Service, Public Health England, London, England; 3 Department of Health Sciences, Global Health, University Medical Centre Groningen, University of Groningen, Groningen, The Netherlands; 4 Healthcare Associated Infection and Antimicrobial Resistance Department, Public Health England, London, England; 5 Department of Infectious Disease Epidemiology, Imperial College London, England; 6 Harvard T.H. Chan School of Public Health, Harvard University, Boston, Massachusetts, United States of America; 7 The Health Foundation, London, England; University of Maryland School of Medicine, UNITED STATES

## Abstract

**Introduction:**

Antibiotic resistance poses a threat to public health and healthcare systems. *Escherichia coli* causes more bacteraemia episodes in England than any other bacterial species. This study aimed to estimate the burden of *E*. *coli* bacteraemia and associated antibiotic resistance in the secondary care setting.

**Materials and methods:**

This was a retrospective cohort study, with *E*. *coli* bacteraemia as the main exposure of interest. Adult hospital in-patients, admitted to acute NHS hospitals between July 2011 and June 2012 were included. English national surveillance and administrative datasets were utilised. Cox proportional hazard, subdistribution hazard and multistate models were constructed to estimate rate of discharge, rate of in-hospital death and excess length of stay, with a unit bed day cost applied to the latter to estimate cost burden from the healthcare system perspective.

**Results:**

14,042 *E*. *coli* bacteraemia and 8,919,284 non-infected inpatient observations were included. *E*. *coli* bacteraemia was associated with an increased rate of in-hospital death across all models, with an adjusted subdistribution hazard ratio of 5.88 (95% CI: 5.62–6.15). Resistance was not found to be associated with in-hospital mortality once adjusting for patient and hospital covariates. However, resistance was found to be associated with an increased excess length of stay. This was especially true for third generation cephalosporin (1.58 days excess length of stay, 95% CI: 0.84–2.31) and piperacillin/tazobactam resistance (1.23 days (95% CI: 0.50–1.95)). The annual cost of *E*. *coli* bacteraemia was estimated to be £14,346,400 (2012 £), with third-generation cephalosporin resistance associated with excess costs per infection of £420 (95% CI: 220–630).

**Conclusions:**

*E*. *coli* bacteraemia places a statistically significant burden on patient health and the hospital sector in England. Resistance to front-line antibiotics increases length of stay; increasing the cost burden of such infections in the secondary care setting.

## Introduction

Ensuring that safe, high quality treatments for infections are available for use is not only important to patients and clinicians, but essential for sustainable health systems. The rising incidence of severe infections (such as bacteraemia), combined with increased drug resistance to treatment options, has been highlighted as a particular risk to human health by World Health Organisation, European Centre for Disease Prevention and Control and the United States (US) Centers for Disease Control and Prevention [1–3]. As a result, there have been a number of national and international initiatives focusing on tackling infections with antibiotic resistance-related pathogens [2,4–6].

*E*. *coli* bacteraemia is a threat to population health internationally. *E*. *coli* bacteraemia incidence has increased in England (from 45.0 cases per 100,000 population in 2010 to 52.0 cases per 100,000 population in 2014) [7], whilst *E*. *coli* is already the most frequent cause of such infections in European hospitals and in community-onset bacteraemia in US elderly patients [8,9]. The threat posed by antibiotic resistant strains of *Escherichia coli* has been highlighted as a particular concern [1–3].

Despite the recent recognition of the public health threat posed by antibiotic resistance generally, and *E*. *coli* bacteraemia in particular, there is limited information as to its economic and health burden [5,10]. Previous studies which have attempted to quantify the health and economic cost associated with *E*. *coli* or Enterobacteriaceae related bacteraemia, have been limited to quantifying the impact of bacteraemia caused by Enterobacteriaceae resistant to third generation cephalosporins from a limited sample of hospitals [8,11], or did not account for patient risk factors and co-morbidities [12]. Evidence from such studies suggests that third generation cephalosporin resistance is significantly associated with increased mortality for patients [8,11]. However, not much is known about the impact of resistance to other commonly used drugs used in bacteraemia treatment pathways, such as piperacillin/tazobactam, ciprofloxacin or gentamicin [7]. Previous evidence of the impact of resistance, in the case of *E*. *coli* or Enterobacteriaceae, also indicates that third-generation cephalosporin resistance or extended-beta lactamase production is associated with increased length of stay (LoS) in hospital [8,11,13]. However, these studies have not been able to utilise national level samples, potentially reducing generalisability of findings and applicability to the national English hospital setting [8,10,11,13]

Previous economic modelling has extrapolated from limited existing estimates to estimate the potential long term impact of infection and resistance [5]. However, there remains a need to produce robust estimates of the current health and economic burden of infection, due to both resistant and sensitive organisms. This will enable evidence-based prioritisation of intervention strategies such as antibiotic stewardship, new antibiotics, vaccines or susceptibility testing [10]. Currently, the national burden of *E*. *coli* bacteraemia (and related resistant infections), in terms of excess mortality and cost, is unknown.

This study aimed to provide robust estimates of the health and cost burden associated with antibiotic resistant and susceptible *E*. *coli* bacteraemia. Firstly, this study aimed to estimate the impact of *E*. *coli* bacteraemia on patient in-hospital mortality and LoS. Secondly, to estimate the effect of antibiotic resistance on these outcomes and thirdly to estimate the annual costs to the English National Health Service (NHS) due to such infections. This study intended to provide estimates that can be utilised in future health economic models, including time-adjusted LoS, cost and in-hospital mortality.

## Materials and methods

### Study design

A retrospective cohort study was performed in the English secondary care setting utilising national whole system datasets. Data cleaning and subsequent statistical analyses were performed in R version 3.3.3, utilising R-packages data.table, survival, etm, mvna and mstate, available at CRAN (available at http://cran.r-project.org) [14–17].

### Study setting, population and outcomes

This study was set in English acute NHS hospitals [18].

### Ethical approval

Approval to utilise pseudo-anonymised data was given by Public Health England in the form of an internal data access agreement, in line with their internal Information Governance procedure. Consent was not obtained as routinely collected data were analysed pseudo-anonymously. Analysis of these data to inform on the epidemiology of bacteria associated with healthcare associated infections and antimicrobial resistance are among the stated purposes for which PHE is authorised to retain and utilise the data used in this work.

#### (i) Exposures of interest

Exposed patients were defined as those who had a record in the *E*. *coli* bacteraemia national mandatory surveillance database between July 1^st^ 2011 and June 30^th^ 2012 [12]. The Public Health England (PHE) mandatory *E*. *coli* bacteraemia surveillance database, for this period, was previously linked deterministically by patient NHS number (a unique patient identifier) with the PHE voluntary susceptibility surveillance database (LabBase2) in a published analysis on 30-day all-cause mortality for *E*. *coli* bacteraemia patients by Abernethy et al. [12]. Abernethy et al. utilised the most recent infection episode if multiple episodes were recorded per patient (though this was only the case for around 5% of patients) [12].

As the *E*. *coli* bacteraemia surveillance is mandatory for all hospitals within England, all laboratory confirmed *E*. *coli* bacteraemia cases should be reported within this dataset. Though susceptibility reporting is voluntary, PHE compared results of sentinel surveillance studies and the LabBase2 system; finding similar patterns in terms of resistance [7]. Because data on hospitalisations were only available for NHS hospitals (see below), only patients admitted to English acute NHS hospitals were included. Note that within the previously-linked laboratory database, 99% of specimens came from acute trust hospitals (according to stated specimen location).

This linked dataset held information on microbiological testing, including antibiotic susceptibility and specimen date, for each *E*. *coli* bacteraemia exposure. In order to estimate additional LoS and mortality attributable to infection, these linked data were further deterministically linked with a national hospital administrative database, ‘Hospital Episode Statistics’ (HES) Admitted Patient Care [19]. This was done using patients’ NHS numbers. Further information on the hospital trust was obtained through deterministic linkage with Estates Return Information Collection [18], using unique provider codes.

Antibiotic resistance, for the purpose of this paper, is defined as *E*. *coli* bacteraemia which has been laboratory-classified as ‘resistant’ and ‘intermediate’ isolates, as done previously with similar datasets [20]. The impact of antibiotic resistance was analysed for resistance to at least one of the following antibiotics: ciprofloxacin, third generation cephalosporins (ceftazidime and/or cefotaxime), gentamicin, piperacillin/tazobactam and carbapenems (imipenem and/or meropenem). In addition, we analysed the impact of resistance against these individual antibiotics, except for carbapenem resistance, which was too rare for individual analysis.

Susceptibility testing was performed at a hospital level, whereby 95% of hospital laboratories use the European Committee on Antimicrobial Susceptibility Testing methodology [20]. “Not tested” in this study refers to cases where there were no relevant susceptibility testing results available for that specific antibiotic (see descriptive statistics presented in the results section for proportions of missingness).

*E*. *coli* bacteraemia isolates that had not been tested for each exposure category were placed into the respective susceptible group. Descriptive statistics indicated that the non-tested isolates were more similar to susceptible than to resistant isolates in terms of crude length of stay, mortality and time-to-event measures. Therefore, throughout this paper ‘susceptible exposed’ refers to *E*. *coli* bacteraemia which has been laboratory-classified as ‘susceptible’ or ‘non-tested’ isolates (unless stated otherwise).

Onset of infection was defined utilising time of specimen as a proxy for time of infection [12]. Community-onset exposed patients were defined as those for which date of specimen was within the first two days of hospital admission [12,20]. If the specimen date was greater than two days post-admission the exposure was classified as hospital-onset.

#### (ii) Non-exposure

“Non-infected” non-exposed patients were defined as all patients who had been admitted to an acute NHS hospital in England (according to HES and estates data [18,21]), with no recorded *E*. *coli* bacteraemia (according to the surveillance database [12]) during that hospital stay. Exposed patients were also treated as non-exposed until the time of infection (time of specimen date).

**Fig 1:**
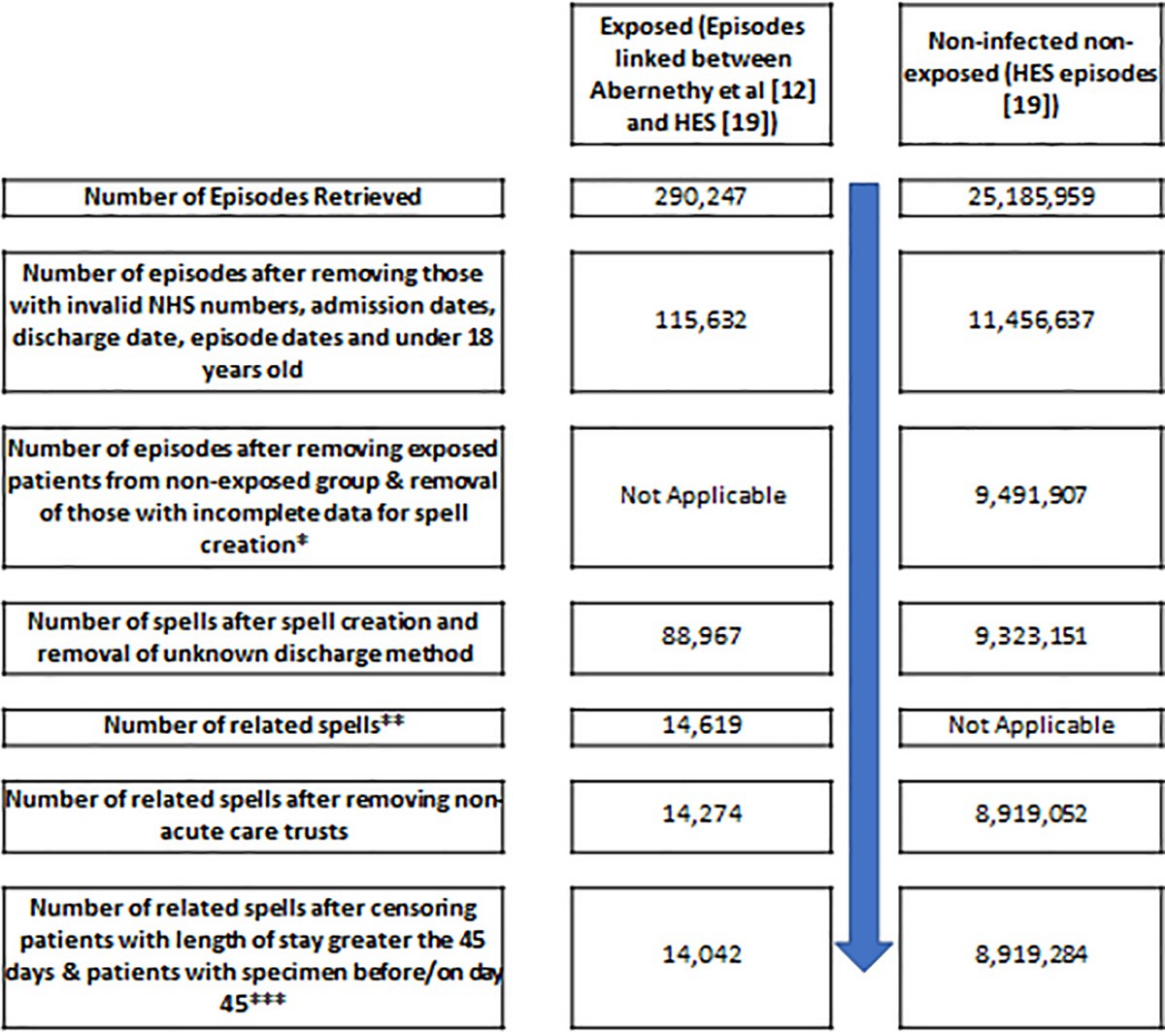
Linked dataset cleaning. *Variables needed for spell creation were HES identifier (‘extract_hesid’), admission date (‘admidate’), provider code, (‘procodet’), start of episode (‘epistart’), end of episode (‘epiend’) and method of discharge (‘dismeth’). **The spell was coded as being related to the exposure if specimen was less 14 days before admission or occurred during admission. *** Those that had not experienced infection prior to or on day 45 were treated as non-exposed patients and censored at day 45 (which causes the move of patients from exposed to unexposed category (n = 232)), this is due to our analysis being performed within the first 45 days of a patient’s hospital spell.

Fig 1 gives an overview of the dataset cleaning and derivation of the final dataset, across exposed and non-exposed cohorts. Exposed and “non-infected” non-exposed patients that had been in hospital longer than 45 days were artificially right-censored, in line with previous work using a similar methodology [11].

#### (iii) Outcomes of interest

The primary outcomes of this study were daily risk of in-hospital mortality (as measured by hazard ratios (HRs) and cumulative incidences) and excess LoS (as measured by HRs and excess days). LoS was defined as the difference between admission date and the last day (discharge or death) of one hospital spell. Same day events (e.g. admission and discharge) were treated as being 0.5 days apart [22].

### Statistical methods

Descriptive statistics were summarised using median and interquartile ranges for continuous variables, and proportions (represented by percentages) for categorical variables.

Previous literature has stated the need to control for time dependency bias and/or competing risks, as well as potential patient/hospital confounders, when investigating the impact of resistance and hospital onset infections on hospital LoS and in-hospital mortality [23,24]. This led to both multistate models (accounting for time-dependency and competing risk bias) and time-dependent hazard models (additionally accounting for patient/hospital covariates) being constructed.

#### (i) In-hospital mortality

When in-hospital mortality is the only available mortality outcome indicator available, the competing risk of being discharged alive should be taken into account [24]. The Aalen-Johansen estimator was used to estimate the cumulative incidence of in-hospital mortality events over the 45-day period, given that patients could also be discharged within that time [15,25]. Pointwise 95% confidence intervals were estimated. Such estimates can be used in the estimation of transition probabilities in health economic models.

However, to investigate whether exposures of interest still significantly impact mortality once other potential covariates were taken into account, subdistribution HRs (SHRs) were calculated [24]. This method puts discharged patients back into the risk-set. The baseline covariates included in the adjusted models were age, sex, a ‘modified Elixhauser comorbidity index’ [26] and hospital trust type [18]. Age and Elixhauser comorbidity index values were centred to their respective means. Infection was treated as a time-dependent exposure [11,22]. Unadjusted and adjusted HRs were then compared to understand the impact of patient covariates on the exposure-outcome relationship.

#### (ii) Length of stay

**Fig 2:**
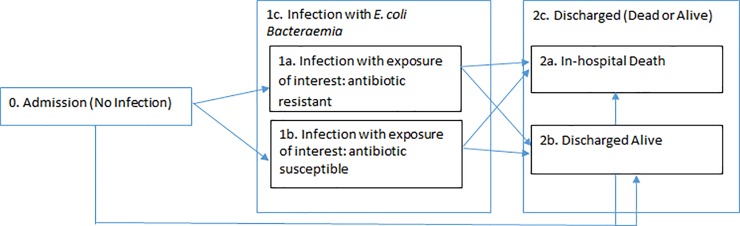
Multistate model schematic for excess length of stay estimation. Potential health states are depicted by the boxes and the direction in which they may travel depicted via arrow directionality. State 0 = “Admission, No Infection”, State 1 = “Infection of Interest” with 1a relating to infections with resistant *E*. *coli*, 1b infections with susceptible *E*. *coli* and composite state 1c being all *E*. *coli* infections, state 2a = “In-hospital Death” and state 2b = “Discharge Alive”. States 2a and 2b, together, form the LoS endpoint, 2c.

To account for time-dependency bias in calculating the impact of exposures on LoS, the Aalen-Johansen estimator was used to estimate the time-varying transition probabilities between the health states within the multistate model [11,25,27]. The Aalen–Johansen estimator is produced from cumulative transition hazards, which are based on the number of patients that are in each state at t-1 and the number of patients that transition out at time t [11, 27]. The expected LoS (on each day), given that you are in a certain state just before this day, was calculated as a function of these transition hazards of moving from the state in question (e.g. not infected) to a discharged state (e.g. discharged alive) [27]. The mean difference in expected LoS between transient states (states 0 and 1a/1b/1c notation in Fig 2) was calculated on each day) and averaged across all days. This method for estimating transition probabilities is in line with previous studies utilising the multistate methodology to estimate infection-related, time-adjusted excess LoS [11,13,22].

The software utilised can construct 3–4 state multistate models [14–17]. Initial multistate models first compared *E*. *coli* infection (1c, Fig 2) to no infection (0, Fig 2) to estimate excess LoS comparing *E*. *coli* exposed to non-infected non-exposed patients(giving *Z*). To estimate the excess LoS comparing resistant and susceptible cases (*Y*), the model was run with the chosen resistant exposure(1a, Fig 2) compared to its respective susceptible exposure (1b, Fig 2), whereby patients entered the model on getting the infection (i.e. enter at 1a or 1b at time of infection, including infection time t = 0). The following equation (which represents a weighted average of excess LoS, weighted by susceptibility prevalence) was then solved;

$$P_{S}*X+P_{R}*(X+Y)=Z$$ (1)

where *P_S_* and *P_R_* represent the proportion of susceptible and resistant cases respectively and *X* represents the excess LoS comparing all susceptible-exposed to non-infected non-exposed. This allowed for the computation of excess LoS estimates for susceptible and resistant exposed patients comparative to non-infected non-exposed.

In the multistate models 95% confidence intervals were estimated through bootstrap sampling, using estimated percentiles.

To investigate whether exposures of interest still significantly impact LoS once other potential covariates were taken into account, Cox proportional hazards models for discharge outcomes were constructed. Adjusted models included the same variables as in the subdistribution HR models for death outcomes.

The proportional hazards assumption was checked utilising plots of corresponding Schoenfeld residuals over time [16]. Slight deviations were found for the infection variable over time, therefore observations were grouped into time periods to account for this, utilising a step function approach, to produce time-dependent coefficients [16]. Therefore, our analysis presents two HRs for each independent variable for different time periods. The HRs for discharge (alive or dead) are defined for the first two days and from two days onwards [16].

Using the above methodology allows for the computation of time- and covariate-adjusted estimates of the impact of independent variables on the daily risk of discharge, a proxy for LoS [11,22]. However, these models do not easily translate into time-adjusted excess LoS in days. Therefore, to attempt to account for time-dependency and patient covariates, whilst providing useful parameters than can be utilised in future health economic models, subgroup analyses were also performed.

#### (iii) Subgroup analysis

Subgroups were defined by age and sex (over and under 65 years of age, for both women and men). In-hospital mortality cumulative incidence and excess LoS were estimated for hospital-onset and community-onset exposed patients separately.

#### (iv) Cost

Costs were estimated from the healthcare system perspective. Cost per spell was estimated by applying England’s Department of Health reference cost for an excess bed-day (an average of bed day cost for days over Health Resource Group ‘limits’) in secondary care (£264 in 2011/12) to the number of excess days estimated in the main analysis [28]. This number was multiplied by the total number of attributable spells (i.e. incidence of attributable hospital spells) to give an estimate of the annual burden in 2011–12. Results were then rounded to the nearest £10 for cost per spell and to the nearest £100 for annual burden, given the lack of certainty surrounding the precision of such estimates. All costs presented are in 2012 Great British Pound (£).

95% confidence intervals were estimated by applying the unit cost to the 95% confidence interval lower and upper bounds for excess LoS estimates. These bounds were then multiplied by incidence to estimate total annual cost 95% confidence intervals.

## Results

Across the period of interest (financial years 2011–2012 inclusive) 25,185,959 hospital episodes were initially retrieved from HES. 290,247 hospital episodes were linked to *E*. *coli* bacteraemia exposed patients. Once the described data cleaning had been applied, 14,042 exposed and 8,919,284 non-exposed spells were included in this study (Fig 1). Tables 1 and 2 show the descriptive statistics of the cohorts analysed, with Table 2 focusing on within-infected characteristics. The majority of cases were community-onset (80%, n = 11,281).

**Table 1:** Descriptive statistics for exposed and non-exposed hospital spells.

Variable	Characteristic (measure)	*E*. *coli* bacteraemia exposed spells	Non-infected Non-exposed spells
Sample Size	Number of Hospital Spells	14,042	8,919,284
s	Male	6,627 (47%)	3,866,241 (43%)
	Female	7,415 (53%)	5,053,034 (56%)
Median Age	Median age (IQR)	75 (62–83)	60 (40–74)
Modified Elixhauser Comorbidity Index	< = 0	5,197 (37%)	5,502,125 (62%)
	1–4	3,415 (24%)	1,483,025 (17%)
	5–9	2,307 (16%)	828,526 (9%)
	10–14	1,939 (14%)	929,340 (10%)
	15–19	740 (5%)	127,550 (1%)
	> = 20	444 (3%)	48,718 (1%)
Organisation Type	Acute (large, medium small)	10,064 (72%)	6,002,799 (67%)
	Other (multi-service, specialist, teaching)	3,978 (28%)	2,916,485 (33%)
Average length of stay	Median days in hospital (IQR)	8 (4–17)	0.5 (0.5–2)
Mortality	In-Hospital Mortality	1,953 (14%)	114,793 (1%)

Descriptive statistics are measured as a count (percentage) unless specified otherwise in the “Characteristic” column. Abbreviations: IQR; interquartile ranges.

**Table 2:** Descriptive statistics by infection type.

Type of Infection		Frequency (%)	In–hospital mortality (%)^a^	Median LoS (IQR)^a^
Onset of Infection	Hospital Onset	2,761 (20%)	588 (21%)	24 (14–39)
	Community Onset	11,281 (80%)	1365 (12%)	6 (3–12)
Third-generation cephalosporin	Susceptible	8,272 (59%)	1127 (14%)	8 (4–16)
	Resistant	879 (6%)	152 (17%)	11 (6–24)
	Not tested or missing	4,891 (35%)	674 (14%)	8 (4–17)
Carbapenem	Susceptible	9,039 (64%)	1265 (14%)	8 (4–17)
	Resistant	11 (0.1%)	1 (9%)	9 (4.5–14)
	Not tested or missing	4,992 (36%)	687 (14%)	8 (4–17)
Ciprofloxacin	Susceptible	8,352 (59%)	1079 (13%)	7 (4–16)
	Resistant	1,803 (13%)	306 (17%)	10 (5–20)
	Not tested or missing	3,887 (28%)	568 (15%)	8 (4–18)
Gentamicin	Susceptible	9,956 (71%)	1361 (14%)	8 (4–16)
	Resistant	924 (7%)	140 (15%)	10 (5–23)
	Not tested or missing	3,162 (23%)	452 (14%)	8 (4–18)
Piperacillin/ tazobactam	Susceptible	8,901 (63%)	1214 (14%)	8 (4–16)
	Resistant	848 (6%)	142 (17%)	12 (6–24)
	Not tested or missing	4,293 (31%)	597 (14%)	8 (4–17)
Resistant to at least one of the above antibiotics	Susceptible to tested antibiotics	8,402 (60%)	1098 (13%)	7 (4–15)
	Resistant to at least one tested antibiotic	2,661 (19%)	428 (16%)	10 (5–20)
	Not tested for any of the above antibiotics	2,979 (21%)	427 (14%)	8 (4–17)

^a^ These are unadjusted descriptive statistics related to each row/characteristic group.

Abbreviations: IQR; interquartile ranges.

### (i) In-hospital mortality

Utilising an Aalen-Johansen estimator to estimate cumulative incidence of in-hospital death, whilst accounting for competing risks, found that 1.29% (95% CI; 1.28%– 1.30%) of non-infected patients experience in-hospital mortality compared to 14.33% (95% CI; 13.74%– 14.94%) of *E*. *coli* bacteraemia patients (S1 Table) within 45 days after admission. Such results show that the 95% CIs do not cross, indicating statistical significance. This is also seen for all tested antibiotic susceptibility exposure groups apart from gentamicin (S1 Table).

Comparing the unadjusted and adjusted subdistribution HR (SHR) estimated utilising a subdistribution hazard approach (Table 3) shows that adjusting for patient and hospital covariates reduces the strength of the association between resistance to all tested antibiotics and in-hospital mortality. Though, *E*. *coli* bacteraemia (regardless of susceptibility profile) still was associated with an increased subdistribution hazard of in-hospital death; almost six-fold.

**Table 3:** Daily subdistribution hazard ratios for in-hospital mortality.

Exposed Group	In-hospital mortality—unadjusted SHR (95% CI)	In-hospital mortality—adjusted SHR (95% CI)^a^
*E*. *coli* bacteraemia	13.27(12.69,13.88)	5.88(5.62,6.15)
*E*. *coli* bacteraemia resistant to at least one tested antibiotic ^b^	16.06(14.61,17.66) †	6.24(5.67,6.86)
*E*. *coli* bacteraemia susceptible to all tested antibiotics ^b^	12.66(12.03,13.31)	5.79(5.50,6.09)
Ciprofloxacin resistant *E*. *coli* bacteraemia	17.96(15.32,21.06)	6.75(5.75,7.91)
Ciprofloxacin susceptible *E*. *coli* bacteraemia	12.99(12.40,13.61)	5.82(5.55,6.10)
Third generation cephalosporin resistant *E*. *coli* bacteraemia	16.93(15.13,18.94) †	6.42(5.74,7.18)
Third generation cephalosporin susceptible *E*. *coli* bacteraemia	12.76(12.16,13.40)	5.79(5.52,6.08)
Gentamicin resistant *E*. *coli* bacteraemia	15.52(13.15,18.32)	6.11(5.18,7.22)
Gentamicin susceptible *E*. *coli* bacteraemia	13.13(12.53,13.75)	5.86(5.60,6.14)
Piperacillin/tazobactam resistant *E*. *coli* bacteraemia	17.7(15.01,20.87) †	7.00(5.93,8.25)
Piperacillin/tazobactam susceptible *E*. *coli* bacteraemia	13.02(12.43,13.64)	5.81(5.54,6.09)

The comparator was “non-infected” non-exposed. Day zero refers to the day of admission.

^a^ Adjusted for age, sex, Elixhauser comorbidity index and organisation type.

^b^ Tested antibiotics included ciprofloxacin, third generation cephalosporins, gentamicin, piperacillin/tazobactam and carbapenems.

† Significant difference between resistant and susceptible cases as defined by confidence intervals.

Abbreviations: CI; confidence interval, SHR; subdistribution hazard ratio.

### (ii) Length of stay

From the multi-state model it was estimated that *E*. *coli* bacteraemia is associated with 3.87 (95% CI; 3.69–4.04) excess hospital days (Table 4). With third generation cephalosporin and piperacillin/tazobactam resistance having the largest association with increased LoS, in comparison to the other tested antibiotics. Third generation cephalosporin was associated with 1.58 (95% CI; 0.84–2.31) excess days compared to equivalent third generation cephalosporin susceptible infections. Ciprofloxacin resistance was not significantly associated with excess LoS in the time adjusted model.

**Table 4:** Excess length of stay estimated by multistate models.

Exposure group	Non-exposed comparator	Excess Length of Stay in Days (95% CI)–Time Adjusted ^a^
*E*. *coli* bacteraemia	“Non-infected” Non-exposed	3.87 (3.69–4.04)
*E*. *coli* bacteraemia resistant to at least one tested antibiotic ^b^	*E*. *coli* bacteraemia susceptible to all tested antibiotics ^b^	0.84 (0.37–2.31) †
Ciprofloxacin resistant *E*. *coli* bacteraemia	Ciprofloxacin susceptible *E*. *coli* bacteraemia	0.46 (-0.11–1.03)
Third generation cephalosporin resistant *E*. *coli* bacteraemia	Third generation cephalosporin susceptible *E*. *coli* bacteraemia	1.58 (0.84–2.31) †
Gentamicin resistant *E*. *coli* bacteraemia	Gentamicin susceptible *E*. *coli* bacteraemia	0.89 (0.20–1.58) †
Piperacillin/tazobactam resistant *E*. *coli* bacteraemia	Piperacillin/tazobactam susceptible *E*. *coli* bacteraemia	1.23 (0.50–1.95) †

Non-infected non-exposed refer to patients in hospital without an *E*. *coli* bacteraemia.

^a^ Time-adjusted through multistate models.

^b^ Tested antibiotics included ciprofloxacin, third generation cephalosporins, gentamicin, piperacillin/tazobactam and carbapenems.

† Significant difference between resistant and susceptible cases as defined by confidence intervals.

Abbreviations: CI; confidence interval.

**Fig 3:**
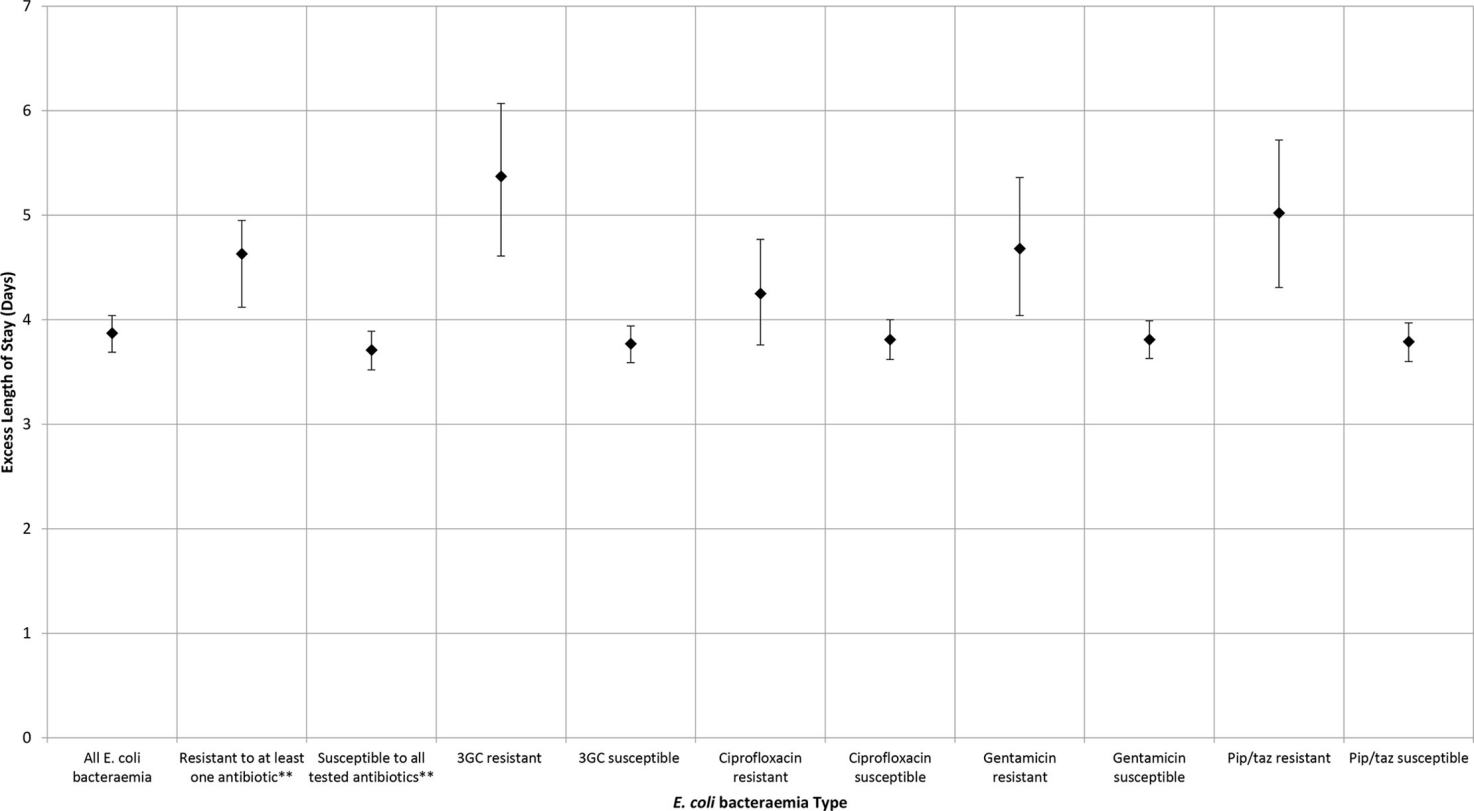
Excess length of Stay of *E*. *coli* Bacteraemia. Excess days associated with an exposure were calculated in comparison to “non-infected” non-exposed, i.e. patients in hospital without an *E*. *coli* bacteraemia. Error bars represent the 95% confidence intervals derived from bootstrapping. **“Resistant to 1 or more” refers to being resistant to at least one of the tested antibiotics (ciprofloxacin, third generation cephalosporins, gentamicin, piperacillin/tazobactam and carbapenems). Abbreviations: 3GC; third-generation cephalosporin, *E*. *coli; Escherichia coli*, pip/taz; piperacillin/tazobactam.

Comparing all exposures of interest to the baseline “non-infected” non-exposed group provided the estimates displayed in Fig 3 (see S2 Table for numerical estimates). This suggests that excess LoS associated with susceptible infections is between three and four days, whilst excess LoS associated with resistant infections is larger, but also more varied and uncertain (dependant on the type of antibiotic resistance).

The Cox proportional hazards models results (Table 5) show that, having accounted for patient characteristics and hospital characteristics on admission, an *E*. *coli* bacteraemia still significantly was associated with a reduced hazard of experiencing a discharge event (alive or dead). Resistance, in all tested, was found to also have a significant association with a reduced hazard of experiencing discharge event (Table 5). Just focusing on those with a LoS of greater than two days from admission, third generation cephalosporin and piperacillin/tazobactam resistance had the strongest association with the hazard of experiencing a discharge event [HR = 0.69 (95% CI; 0.64–0.74) for both] (Table 5). This is consistent with the multistate model results.

**Table 5:** Daily risk of experiencing discharge events.

Exposure group	Adjusted HR for the first two days (95% CI) ^a^	Adjusted HR from two days onwards (95% CI) ^a^
*E*. *coli* bacteraemia	0.15 (0.15–0.16)	0.83 (0.82–0.84)
*E*. *coli* bacteraemia resistant to at least one tested antibiotic ^b^	0.14 (0.13–0.15)	0.75 (0.72–0.78) †
*E*. *coli* bacteraemia susceptible to all antibiotics tested^b^	0.16 (0.15–0.16)	0.85 (0.83–0.86)
Third generation cephalosporin resistant *E*. *coli* bacteraemia	0.12 (0.10–0.14) †	0.69 (0.64–0.74) †
Third generation cephalosporin susceptible *E*. *coli* bacteraemia	0.16 (0.15–0.16)	0.84 (0.82–0.85)
Ciprofloxacin resistant *E*. *coli* bacteraemia	0.14 (0.12–1.16)	0.76 (0.72–0.80) †
Ciprofloxacin susceptible *E*. *coli* bacteraemia	0.16 (0.15–0.16)	0.84 (0.82–0.86)
Gentamicin resistant *E*. *coli* bacteraemia	0.12 (0.10–0.14) †	0.75 (0.70–0.80) †
Gentamicin susceptible *E*. *coli* bacteraemia	0.16 (0.15–0.16)	0.83 (0.82–0.85)
Piperacillin/tazobactam resistant *E*. *coli* bacteraemia	0.13 (0.11–0.16)	0.69 (0.64–0.74) †
Piperacillin/tazobactam susceptible *E*. *coli* bacteraemia	0.15 (0.14–0.17)	0.83 (0.82–0.85)

Cause-specific hazard ratios for time to discharge (alive or dead) estimated through Cox proportional hazards models, the comparator was “non-infected” non-exposed. Day zero refers to the day of admission.

^a^ Adjusted for age, sex, Elixhauser comorbidity index and organisation type.

^b^ Tested antibiotics included ciprofloxacin, third generation cephalosporins, gentamicin, piperacillin/tazobactam and carbapenems.

† Significant difference between resistant and susceptible cases as defined by confidence intervals.

Abbreviations: CI; confidence interval, HR; hazard ratio.

### (iii) Subgroup analysis

To provide age and sex-adjusted estimates of LoS (in days) and incidence of mortality (in terms of cumulative incidence at day 45), the same multistate model methods utilised above were performed on subgroups. *E*. *coli* bacteraemia was associated with in-hospital mortality and excess LoS in all subgroups. Resistance was not clearly associated with the cumulative incidence of in-hospital mortality in most subgroups tested for most antibiotic susceptibilities (see Table 6 and S3 Table). This is consistent with the subdistribution hazard results.

**Table 6:** Subgroup analyses of in-hospital mortality and length of stay outcomes.

Subgroup (Sex, Age)	Exposure	Sample size	Cumulative Incidence of In-hospital Death at Day 45 % (95% CI)	Excess Length of Stay Compared to Non-Infected Non-exposed Days (95% CI)
*Escherichia coli* bacteraemia subgroup analyses				
Males, 18–64	Non-infected non-exposed	1,983,009	0.44 (0.43,0.45)	-
	*E*. *coli* Bacteraemia	1,708	12.01 (10.48,13.75) †	4.54 (4.07,5.02) †
Females, 18–64	Non-infected non-exposed	3,196,801	0.21 (0.21,0.22)	-
	*E*. *coli* Bacteraemia	2,336	6.88 (5.91,8.00) †	3.75 (3.38,4.08) †
Males, 65+	Non-infected non-exposed	1,883,117	2.53 (2.51,2.55)	
	*E*. *coli* Bacteraemia	4,919	17.28 (16.21,18.41) †	2.48 (2.22,2.78) †
Females, 65+	Non-infected non-exposed	1,856,125	2.78 (2.76,2.81)	-
	*E*. *coli* Bacteraemia	5,079	15.86 (14.85,16.94) †	2.26 (1.96,2.58) †
Third general cephalosporin susceptibility subgroup analyses				
Males, 18–64	Resistant	124	13.15 (8.01,21.17)	5.22 (3.54,6.92)
	Susceptible	124	11.93 (10.35,13.73)	4.48 (3.97,5.00)
Females, 18–64	Resistant	113	15.06 (9.28,23.92) †	4.50 (2.94,6.08)
	Susceptible	2,223	6.50 (5.54,7.62)	3.71 (3.36,4.04)
Males, 65+	Resistant	320	25.55 (20.82,31.12) †	3.96 (2.69,5.23)
	Susceptible	4,599	16.75 (15.66,17.90)	2.38 (2.10,2.68)
Females, 65+	Resistant	322	15.31 (11.65,19.97)	3.89 (2.67,4.99)
	Susceptible	4,757	15.90 (14.85,17.01)	2.15 (1.84,2.47)
Piperacillin/tazobactam susceptibility subgroup analyses				
Males, 18–64	Resistant	105	10.04 (5.33,18.46)	5.13 (3.35,6.85)
	Susceptible	1,603	12.13 (10.55,13.93)	4.50 (4.02,5.01)
Females, 18–64	Resistant	113	11.95 (6.94,20.16)	4.75 (2.85,6.73)
	Susceptible	2,223	6.64 (5.67,7.77)	3.69 (3.32,4.07)
Males, 65+	Resistant	337	24.70 (20.10,30.15) †	3.68 (2.48,4.83)
	Susceptible	4,582	16.80 (15.71,17.95)	2.39 (2.12,2.72)
Females, 65+	Resistant	293	17.16 (13.07,22.36)	3.37 (2.31,4.52)
	Susceptible	4,786	15.79 (14.75,16.89)	2.19 (1.89,2.50)

† Significant difference as defined by confidence intervals.

Abbreviations: CI; confidence interval.

Third-generation cephalosporin resistance and piperacillin/tazobactam resistance were the only individual antibiotic resistance profiles that were associated with LoS outcomes when subgroups were tested. Results for these antibiotic susceptibility profiles are provided in Tables 6. Comparing results across the different subgroup analyses presented in Tables 6 highlights that having an *E*. *coli* bacteraemia was associated with stronger effects among younger subgroups, whilst resistance was associated with larger effects among the older subgroups, in terms of average excess length of stay. For other subgroup estimates related to other antibiotic exposure groups of interest refer to S3 Table.

Community-onset cases had substantially larger estimates of excess LoS [3.32 days (95% CI; 3.13–3.51)] compared to hospital-onset cases [0.96 days (95% CI; 0.84–1.08)]. Meanwhile, hospital-onset cases had a significantly higher in-hospital mortality incidence. In-hospital death cumulative incidence was 30.80% (95% CI; 28.02–33.79%) for hospital-onset versus 12.10% (95% CI; 11.51–12.72%) for community onset.

### (iv) Cost

Applying the Department of Health Reference cost for an excess bed-day [28] to the estimates for excess LoS gives a cost per spell of per in-patient with *E*. *coli* bacteraemia of £1,020 (95% CI;£970 –£1,070). Utilising this cost per spell and number of spells, the estimated annual cost burden to hospitals due to *E*. *coli* bacteraemia in 2011/12 was £14,346,400 (Table 7). Adjusting only for time dependency bias, excess annual costs associated with third generation cephalosporin resistance and piperacillin/tazobactam (comparative to if these had been susceptible infections) were £366,600 (95% CI; £194,927 –£550,000) and £275,400 (95% CI; £105,200 - £436,600) respectively (Table 7). That is to say, if all third generation cephalosporin resistant infections had been susceptible it was estimated that £366,600 would not have been spent on those infections (based on reduced LoS).

**Table 7:** Excess costs associated with *E*. *coli* bacteraemia.

Exposure of Interest	Incremental Excess Cost in 2012 £s (95% CI) ^a^	Excess Annual Cost in 2012 £s (95% CI) ^b^
Compared to ‘Non-infected’ Non-exposed		
*E*. *coli* Bacteraemia	1,020 (970–1,070)	14,346,400 (13,679,200–14,976,700)
Compared to Susceptible Infections		
*E*. *coli* bacteraemia resistant to at least one tested antibiotic***	220 (100–340)	583,100 (274,000–906,200)
Third Generation Cephalosporin resistant *E*. *coli* bacteraemia	420 (220–630)	366,600 (194,927–550,000)
Ciprofloxacin resistant *E*. *coli* bacteraemia	120 (-30–270)	219,000 (-47,600–490,300)
Gentamicin resistant *E*. *coli* bacteraemia	230 (60–410)	217,100 (51,200–378,100)
Piperacillin/tazobactam susceptible *E*. *coli* bacteraemia	320 (120–510)	275,400 (105,200–436,600)

Costs and 95% confidence intervals were derived applying a unit cost to estimates of excess LoS.

^a^Rounded to the nearest £10

^b^Rounded to the nearest £100.

Abbreviations: £; Great British Pound, CI; confidence interval.

***Tested antibiotics included ciprofloxacin, third generation cephalosporins, gentamicin, piperacillin/tazobactam and carbapenems.

## Discussion

This study estimated that *E*. *coli* bacteraemia (both resistant and susceptible) is significantly associated with a higher daily risk of experiencing in-hospital mortality, with time- and covariate-adjusted *E*. *coli* bacteraemia exposure being associated with a SHR of 5.88 (95% CI; 5.62,6.15). *E*. *coli* bacteraemia places a substantial cost burden on NHS hospitals, being associated with an excess LoS of almost four days per infection and an annual cost of over £14 million (time-adjusted).

Though resistance to most antibiotics (tested in this analysis) was associated with a significantly increased cumulative incidence of in-hospital mortality when tested on the full sample, this result did not hold in subgroup analyses. Across the different models utilised, resistance to either piperacillin/tazobactam or to third generation cephalosporin was found to have a significant impact on LoS, and therefore estimated costs. Third generation cephalosporin resistance was estimated to have an excess cost of over £350,000 between July 2011 –June 2012, when just accounting for time dependency bias.

### Comparison with previous research

A study estimating the impact of third generation cephalosporin resistant and susceptible Enterobacteriaceae bloodstream infections in Europe found susceptible infections (compared to “non-infected”) to have an excess LoS of 4.36 days (95% CI; 3.91–4.81) [11]. This is similar to our estimate of 3.71 days (95% CI; 3.29–4.13) for any *E*. *coli* bacteraemia. However, the impact of resistance in the European study was higher than in ours. We estimated resistance to third generation cephalosporins to increase LoS (comparative to susceptible controls) by 1.58 (95% CI; 0.84–2.31) days, compared to their estimate of 3.53 days (95% CI; 2.08–4.96) [11]. However, this could partly be due to the lower reliance on third generation cephalosporins in England than in Europe [29,30], though the impact of all other antibiotic resistances (such as piperacillin/tazobactam) was still less than 3 days in our analysis. This could also be due to the inclusion of “non-tested” into our susceptible group, which could mean our results are conservative. This is the first study estimating the impact of other antibiotic resistances on LoS (with regards to *E*. *coli* bacteraemia), such as piperacillin/tazobactam or ciprofloxacin, whilst adjusting for time dependency and patient characteristics. Ciprofloxacin was found to not have a significant impact on LoS across the multistate model analyses. One reason for this observation could be the reduced usage of such fluoroquinolones in England, in an attempt to reduce *Clostridium difficile* infections [31].

In terms of impact on mortality, when adjusting for competing risks and patient covariates (utilising subdistribution hazards and subgroup cumulative incidence of in-hospital mortality), a significant impact of resistance was not generally seen. This is not in agreement with a comparable European study on *Enterobacteriaceae*-related bacteraemia, which found third generation cephalosporin resistance to have a significant impact [HR = 1.63 (95% CI: 1.13–2.35)] [11]. This again could be due to a lower reliance on such antibiotics in England [29,30], or due to reduced study power in the subgroup analyses. Our results are in agreement with an earlier European study, which found such resistance in *E*. *coli* bacteraemia was not significantly associated with mortality [HR = 2.5 (95% CI; 0.9–6.8)] [8], though the cited study had limited power to detect a true effect as evidenced by the wide confidence intervals. A study on 30-day all-cause mortality utilising similar data-sources found that ciprofloxacin did have a significant impact on mortality, whereas our analysis did not find ciprofloxacin resistance to be significantly associated with in-hospital mortality [12]. This may be as our results adjust for patient comorbidity, something the aforementioned study was unable to do due to data limitations [12]. Our adjustment for patient comorbidity (the modified Elixhauser comorbidity index [26]) was dependent on ICD-10 codes for the index hospitalization, which may therefore include conditions which developed post-bacteraemia episode. However, this risk was reduced by only utilising the codes stated in the initial Finished Consultant Episode of a patient’s hospital spell.

### Implications

*E*. *coli* bacteraemia was found in this study to cause a considerable cost burden in terms of excess LoS. This, in addition to the increase in the incidence in *E*. *coli* bacteraemia seen over the past few years and the recent policy to attempt to reduce Gram-negative bacteraemia in NHS hospitals [6,32], would suggest evidence-based national bacteraemia treatment guidance may be beneficial. The study showed that it was not just resistant infections that had a large cost impact, but that antibiotic susceptible *E*. *coli* bacteraemia are associated with substantial excess costs too. This would indicate that there is a need to tackle *E*. *coli* bacteraemia in general, not just focus on reducing resistant infections.

While there has been investment through initiatives to encourage infection control in the hospital setting [4–6], in this study hospital-onset infections accounted for less than a quarter of all *E*. *coli* bacteraemia cases, suggesting purely hospital focused initiatives may be limited in their impact. In addition, subgroup analysis suggests that community onset infections are associated with a larger burden in terms of excess LoS. This would indicate that policies to improve the treatment of less serious infections (such as urinary tract infections) arising in the community that can develop into bacteraemia [20] could be something to be further investigated in terms of reducing the cost burden of *E*. *coli* bacteraemia. However, it should be noted that community-onset healthcare-associated infections were not distinguished from community-onset community-associated infections when estimating the impact of such infections.

### Strengths and limitations

Our study is the first to estimate excess LoS associated with antibiotic resistant bloodstream infections utilising a multistate model methodology and a nationally representative dataset, providing a large sample size of over 8 million non-exposed spells and over 14,000 exposed spells. It provides the first national estimate on the annual cost burden of *E*. *coli* bacteraemia. Our study accounts for time-dependency bias and additional covariates in estimating excess LoS. Both the Cox models and multistate models are in agreement that piperacillin/tazobactam and third generation are associated with the largest impact on time to discharge/excess LoS, with ciprofloxacin estimated to have either the least or a non-significant impact. This agreement across different models (with different structures) reduces the likelihood that such conclusions due to type one error.

One limitation of this study is that it is retrospective in nature and a cohort study based on data collected for other purposes, meaning that some data used may have been wrongly coded, missing or skewed. For example, the laboratory data collected on resistance profiles was from a voluntary database, meaning certain institutions with certain characteristics may more commonly report these isolate results. However, PHE has stated within national surveillance reports that the susceptibility surveillance data reported from LabBase2 are nationally representative, with an ascertainment rate of over 84% (in relation to mandatory *E*. *coli* surveillance) in 2012–2013 [7, 32]. Our analysis did not include antibiotic exposure, since data were not available, though did account for timing of infection through time dependent covariates and multistate methodology [16,27].

A further limitation of our study is that we cannot exclude the possibility that our results, despite adjustment for patient demographics and comorbidities, are overestimating the impact of *E*. *coli* bacteraemia on in-hospital mortality and LoS. For example, we did not have information about potential time-dependent confounders that could confound the association between hospital-acquired bacteraemia and in-hospital mortality and length of stay, such as the evolution of underlying severity of illness or invasive producers. If such data would be available, g-methods like marginal structural models with inverse probability weighting could be used to adjust for potential time-dependent confounders.[33]. It should also be reiterated that the results of this analysis are only related to excess hospital days and deaths occurring during the first 45 days of patient’s stay. However, this captures the vast majority of infections, and aligns this research with previous work [11].

Inherently the multistate model methodology only accounts for time dependency bias and does not correct for patient or hospital characteristics, however, subgroup analyses were performed to provide estimates in relation to different sample populations. Though subgroup analyses provide useful estimates in clinical practice, this led to smaller sample sizes and thus reduced statistical power. Nonetheless, in all tests the number of resistant cases was above that of 100, providing similar numbers of resistance cases as utilised in previous estimations of antimicrobial resistance hospital burden [8,11].

Place of onset was also compared utilising subgroup analysis, however the cut-off for inclusion in each category (2 days post-admission for inclusion in hospital-onset) is arbitrary and may incorporate bias in its current form. In addition, there is no analysis in trends over time, as the scale of this analysis is limited to one year (July 2011 –June 2012). However, until the 2016/17 incentives to reduce Gram-negative bloodstream infections there were no national changes in governmental recommendations for the treatment of *E*. *coli* bloodstream infections [34], meaning that the estimates should be generalizable outside the years in the study cohort. Carbapenem resistance was not investigated individually, given that there were only 11 cases in our sample, though was included as a potential resistance for the “resistant to one or more of the tested antibiotics” group. Carbapenem resistance is a major cause for concern [35], and investigation into the current and potential future impact of such resistance is needed.

The impact of resistance on mortality, LoS and subsequent costs was estimated by grouping tested-susceptible and non-tested subjects. This approach may have introduced bias, as those not-tested could theoretically be resistant, however descriptive and time-to-event statistics were utilised to verify this assumption. Bias introduced in this sense would mean our estimates are likely conservative. The costing does not account for any additional drug costs, procedural costs, ‘infection prevention and control’ costs, or costs due to subsequent treatment in the community or readmission to hospital, meaning our estimates are likely conservative. However, recent literature suggests that LoS is a key cost factor when estimating the costs of hospital-onset infections [23], meaning our estimates likely account for a key proportion of costs. Our costing is an average unit cost of a hospital bed day and applied to all admission types, in reality a patient who was in intensive care could likely cost much more. Co-resistance was not taken into account within our analysis, since each infection type was analysed in the Cox and multistate models separately, to reduce computational complexity. Levels of co-resistance have remained stables over recent years within such infections [36].

Furthermore, in-hospital all-cause mortality is utilised as an outcome rather than total attributable mortality or 30-day mortality. Further data linkage for both cases and non-exposed to the office of national statistics mortality dataset would allow the full impact of infection on mortality, utilising Cox proportional hazards models, to be investigated in future research.

## Conclusions

The growing incidence of serious bacterial infections and the prevalence of antibiotic resistance are a threat to both patients and healthcare systems. Our findings quantify the cost and mortality burden of *E*. *coli* bacteraemia and the influence of different resistances on this; estimating an annual excess cost of over £14 million and over £500,000 for *E*. *coli* bacteraemia and ‘resistance to at least one tested antibiotic’ respectively. Such findings will be useful for those identifying priorities for investment, infection control and modelling the health and economic impact of future trends in resistance. Additional research is needed to identify modifiable health system and wider factors which could improve the outcomes of patients with and susceptible *E*. *coli* bacteraemia, to aid the development of effective interventions aimed at reducing such infections. Our findings suggest such interventions could potentially improve care for patients while potentially reducing health system costs, though dependent on intervention effectiveness and costs.

## Supporting information

S1 Table Cumulative incidence of in-hospital mortality. Results of cumulative incidence of mortality functions by day 45.
(DOCX)

S2 Table Excess length of stay in comparison to “non-infected” non-exposed. Additional length of stay results from multistate models.
(DOCX)

S3 Table Excess length of stay in subgroup analysis. Additional length of stay results from multistate models.
(DOCX)

## Abbreviations

CI: confidence interval
HES: Hospital Episode Statistics
HR: hazard ratio
LoS: length of stay
NHS: National Health Service
PHE: Public Health England
SHR: subdistribution hazard ratio
US: United States
