# Supplementary material for: The health and cost burden of antibiotic resistant and susceptible Escherichia coli bacteraemia in the English hospital setting: A national retrospective cohort study
Source: PLoS One. 2019 Sep 10;14(9):e0221944. doi: 10.1371/journal.pone.0221944 (PMC6736296; doi:10.1371/journal.pone.0221944)
Supplement: S1 Table — Results of cumulative incidence of mortality functions by day 45. (DOCX) [file pone.0221944.s001.docx]

**S1 Table. Cumulative Incidence of In-hospital Mortality.** Results present the cumulative incidence of mortality by day 45, estimated using the Aalen-Johansen estimator to account for the competing risk of discharge. *Tested antibiotics included ciprofloxacin, third generation cephalosporins, gentamicin, piperacillin/tazobactam and carbapenems.

| **Cohort** | **Cumulative Incidence % (95% CIs)** |
| --- | --- |
| Non-infected controls | 1.29 (1.28,1.30) |
| *E. coli* bacteraemia | 14.33 (13.74,14.94) |
| *E. coli* bacteraemia resistant to at least one tested antibiotic* | 16.91 (15.47,18.47) |
| *E. coli* bacteraemia susceptible to all tested antibiotics* | 13.76 (13.12,14.43) |
| Ciprofloxacin resistant *E. coli* bacteraemia | 18.71 (16.12,21.65) |
| Ciprofloxacin susceptible *E. coli* bacteraemia | 14.07 (13.46,14.69) |
| Third generation cephalosporin resistant *E. coli* bacteraemia | 17.72 (15.96,19.66) |
| Third generation cephalosporin susceptible *E. coli* bacteraemia | 13.84 (13.22,14.49) |
| Gentamicin resistant *E. coli* bacteraemia | 15.90 (13.56,18.59) |
| Gentamicin susceptible *E. coli* bacteraemia | 14.23 (13.62,14.86) |
| Piperacillin/tazobactam resistant *E. coli* bacteraemia | 18.62 (15.97,21.64) |
| Piperacillin/tazobactam susceptible *E. coli* bacteraemia | 14.09 (13.49,14.72) |
