# Supplementary material for: The health and cost burden of antibiotic resistant and susceptible Escherichia coli bacteraemia in the English hospital setting: A national retrospective cohort study
Source: PLoS One. 2019 Sep 10;14(9):e0221944. doi: 10.1371/journal.pone.0221944 (PMC6736296; doi:10.1371/journal.pone.0221944)
Supplement: S2 Table — Additional length of stay results from multistate models. (DOCX) [file pone.0221944.s002.docx]

**S2 Table. Excess Length of Stay in Comparison to “Non-Infected” Non-Exposed**

Estimated utilising multistate models *Tested antibiotics included ciprofloxacin, third generation cephalosporins, gentamicin, piperacillin/tazobactam and carbapenems.

| **Exposure Group** | **Excess Length of Stay**  **(95% Confidence Interval)** |
| --- | --- |
| *E. coli* bacteraemia | 3.87 (3.69,4.04) |
| *E. coli* bacteraemia resistant to at least one tested antibiotic* | 4.63 (4.12,4.95) |
| *E. coli* bacteraemia susceptible to all tested antibiotics* | 3.71 (3.52,3.89) |
| 3GC resistant *E. coli* bacteraemia | 5.37 (4.61,6.07) |
| 3GC susceptible *E. coli* bacteraemia | 3.77 (3.59,3.94) |
| Ciprofloxacin resistant *E. coli* bacteraemia | 4.25 (3.76,4.77) |
| Ciprofloxacin susceptible *E. coli* bacteraemia | 3.81 (3.62,4.00) |
| Gentamicin resistant *E. coli* bacteraemia | 4.68 (4.04,5.36) |
| Gentamicin susceptible *E. coli* bacteraemia | 3.81 (3.63,3.99) |
| Piperacillin/tazobactam resistant *E. coli* bacteraemia | 5.02 (4.31,5.72) |
| Piperacillin/tazobactam susceptible *E. coli* bacteraemia | 3.79 (3.60,3.97) |
