# Supplementary material for: The health and cost burden of antibiotic resistant and susceptible Escherichia coli bacteraemia in the English hospital setting: A national retrospective cohort study
Source: PLoS One. 2019 Sep 10;14(9):e0221944. doi: 10.1371/journal.pone.0221944 (PMC6736296; doi:10.1371/journal.pone.0221944)
Supplement: S3 Table — Additional length of stay results from multistate models. (DOCX) [file pone.0221944.s003.docx]

**S3 Table. Excess Length of Stay in Subgroup Analyses**

* As estimated by cumulative incidence within 45 days. **Tested antibiotics included Ciprofloxacin, third generation cephalosporins, gentamicin, piperacillin/tazobactam and carbapenems. †Resistance was associated with a significant impact, as defined by comparing 95 % confidence intervals. Abbreviations: 3GC; Third-generation cephalosporin, CI; confidence interval.

| **Subgroup (Gender, Age)** | **Exposure** | **Sample size** | **Cumulative Incidence of In-hospital Death at Day 45***  **% (95% CI)** | **Excess Length of Stay Compared to Non-Infected Controls***  **Days (95% CI)** | **Excess Length of Stay Compared to Susceptible *E. coli* bacteraemia**  **Days (95% CI)** |
| --- | --- | --- | --- | --- | --- |
| Males,  18-64 | Non-infected non-exposed | 1,983,009 | 0.44 (0.43,0.45) | - | - |
|  | *E. coli* Bacteraemia | 1,708 | 12.01 (10.48,13.75) | 4.54 (4.07,5.02) | - |
|  | Resistant to ≥1 tested antibiotic** | 356 | 10.39 (7.46,14.38) | 4.80 (3.73,5.83) | 0.33 (-0.82,1.56) |
|  | Susceptible to all tested antibiotics** | 1,352 | 12.43 (10.7,14.41) | 4.47 (3.94,5.01) | - |
|  | 3GC resistant | 124 | 13.15 (8.01,21.17) | 5.22 (3.54,6.92) | 0.73 (-0.98,2.52) |
|  | 3GC susceptible | 124 | 11.93 (10.35,13.73) | 4.48 (3.97,5) |  |
|  | Ciprofloxacin resistant | 243 | 10.43 (6.97,15.47) | 4.44 (3.05,5.79) | -0.11 (-1.62,1.42) |
|  | Ciprofloxacin susceptible | 1,465 | 12.29 (10.63,14.18) | 4.55 (4.04,5.07) | - |
|  | Gentamicin resistant | 125 | 10.57 (5.9,18.54) | 4.06 (2.34,6.04) | -0.51 (-2.31,1.45) |
|  | Gentamicin susceptible | 1,583 | 12.14 (10.55,13.95) | 4.58 (4.08,5.07) | - |
|  | Piperacillin/tazobactam resistant | 105 | 10.04 (5.33,18.46) | 5.13 (3.35,6.85) | 0.63 (-1.21,2.32) |
|  | Piperacillin/tazobactam susceptible | 1,603 | 12.13 (10.55,13.93) | 4.50 (4.02,5.01) | - |
| Females,  18-64 | Non-infected controls | 3,196,801 | 0.21 (0.21,0.22) | - | - |
|  | *E. coli* Bacteraemia | 2,336 | 6.88 (5.91,8.00) | 3.75 (3.38,4.08) | - |
|  | Resistant to ≥1 tested antibiotic | 336 | 11.75 (8.63,15.89) † | 4.18 (3.11,5.24) | 0.51 (-0.74,1.71) |
|  | Susceptible to all tested antibiotics | 2,000 | 6.1 (5.13,7.26) | 3.67 (3.27,4.03) | - |
|  | 3GC resistant | 113 | 15.06 (9.28,23.92) † | 4.50 (2.94,6.08) | 0.79 (-0.83,2.41) |
|  | 3GC susceptible | 2,223 | 6.5 (5.54,7.62) | 3.71 (3.36,4.04) | - |
|  | Ciprofloxacin resistant | 202 | 11.5 (7.67,17.07) † | 4.75 (3.38,6.19) | 1.1 (-0.4,2.61) |
|  | Ciprofloxacin susceptible | 2,134 | 6.48 (5.5,7.62) | 3.65 (3.26,4.01) | - |
|  | Gentamicin resistant | 127 | 7.62 (4.13,13.86) | 4.01 (2.37,5.62) | 0.28 (-1.37,2.03) |
|  | Gentamicin susceptible | 2,209 | 6.83 (5.84,7.98) | 3.73 (3.36,4.07) | - |
|  | Piperacillin/tazobactam resistant | 113 | 11.95 (6.94,20.16) | 4.75 (2.85,6.73) | 1.06 (-0.83,3.11) |
|  | Piperacillin/tazobactam susceptible | 2,223 | 6.64 (5.67,7.77) | 3.69 (3.32,4.07) | - |
| Males,  65+ | Non-infected controls | 1,883,117 | 2.53 (2.51,2.55) |  | - |
|  | *E. coli* Bacteraemia | 4,919 | 17.28 (16.21,18.41) | 2.48 (2.22,2.78) | - |
|  | Resistant to ≥1 tested antibiotic | 1,033 | 20.91 (18.45,23.64) † | 2.99 (2.28,3.69) | 0.65 (-0.1,1.46) |
|  | Susceptible to all tested antibiotics | 3,886 | 16.34 (15.18,17.59) | 2.35 (2.03,2.69) | - |
|  | 3GC resistant | 320 | 25.55 (20.82,31.12) † | 3.96 (2.69,5.23) | 1.58 (0.28,2.87)† |
|  | 3GC susceptible | 4,599 | 16.75 (15.66,17.90) | 2.38 (2.1,2.68) | - |
|  | Ciprofloxacin resistant | 740 | 21.44 (18.55,24.72) † | 2.54 (1.73,3.31) | 0.07 (-0.83,0.88) |
|  | Ciprofloxacin susceptible | 4,179 | 16.55 (15.41,17.76) | 2.47 (2.17,2.81) | - |
|  | Gentamicin resistant | 348 | 21.05 (16.87,26.10) | 3.48 (2.33,4.66) | 1.08 (-0.16,2.28) |
|  | Gentamicin susceptible | 4,571 | 17 (15.91,18.17) | 2.41 (2.13,2.73) | - |
|  | Piperacillin/tazobactam resistant | 337 | 24.70 (20.10,30.15) † | 3.68 (2.48,4.83) | 1.28 (0.05,2.46) † |
|  | Piperacillin/tazobactam susceptible | 4,582 | 16.80 (15.71,17.95) | 2.39 (2.12,2.72) | - |
| Females,  65+ | Non-infected controls | 1,856,125 | 2.78 (2.76,2.81) | - | - |
|  | *E. coli* Bacteraemia | 5,079 | 15.86 (14.85,16.94) | 2.26 (1.96,2.58) | - |
|  | Resistant to ≥1 tested antibiotic | 936 | 16.82 (14.46,19.52) | 3.1 (2.43,3.83) | 1.04 (0.29,1.83) † |
|  | Susceptible to all tested antibiotics | 4,143 | 15.65 (14.55,16.83) | 2.06 (1.73,2.41) | - |
|  | 3GC resistant | 322 | 15.31 (11.65,19.97) | 3.89 (2.67,4.99) | 1.74 (0.57,2.92) † |
|  | 3GC susceptible | 4,757 | 15.90 (14.85,17.01) | 2.15 (1.84,2.47) | - |
|  | Ciprofloxacin resistant | 618 | 18.15 (15.20,21.60) | 2.73 (1.83,3.56) | 0.54 (-0.39,1.47) |
|  | Ciprofloxacin susceptible | 4,461 | 15.54 (14.47,16.68) | 2.19 (1.88,2.52) | - |
|  | Gentamicin resistant | 324 | 16.06 (12.29,20.84) | 3.3 (2.09,4.41) | 1.11 (-0.14,2.28) |
|  | Gentamicin susceptible | 4,755 | 15.85 (14.81,16.96) | 2.19 (1.89,2.51) | - |
|  | Piperacillin/tazobactam resistant | 293 | 17.16 (13.07,22.36) | 3.37 (2.31,4.52) | 1.19 (0.07,2.39) † |
|  | Piperacillin/tazobactam susceptible | 4,786 | 15.79 (14.75,16.89) | 2.19 (1.89,2.50) | - |
